# Nutrition and cognitive health: A life course approach

**DOI:** 10.3389/fpubh.2023.1023907

**Published:** 2023-03-27

**Authors:** Seema Puri, Majida Shaheen, Bhanvi Grover

**Affiliations:** Department of Food and Nutrition, Institute of Home Economics, University of Delhi, New Delhi, India

**Keywords:** diet, cognitive health, dementia, healthy aging, nutrient, life course approach

## Abstract

Multiple factors affect cognitive health, such as age-related changes in the brain, injuries, mood disorders, substance abuse, and diseases. While some cannot be changed, evidence exists of many potentially possibly modifiable lifestyle factors: diet, physical activity, cognitive and social engagement, smoking and alcohol consumption which may stabilize or improve declining cognitive function. In nutrition, the focus has been mainly on its role in brain development in the early years. There is a strong emerging need to identify the role of diet and nutrition factors on age-related cognitive decline, which will open up the use of new approaches for prevention, treatment or management of age-related disorders and maintaining a good quality of life among older adults. While data on effect of high protein diets is not consistent, low-fat diets are protective against cognitive decline. Several micronutrients like B group vitamins and iron, as well as many polyphenols play a crucial role in cognitive health. Mediterranean, Nordic, DASH, and MIND diets are linked to a lower risk of cognitive decline and dementia. The relationship between the gut microbiome and brain function through the gut-brain axis has led to the emergence of data on the beneficial effects of dietary fibers and probiotics through the management of gut microbes. A “whole diet” approach as well as macro- and micro-nutrient intake levels that have protective effects against cardiovascular diseases are most likely to be effective against neurodegenerative disorders too. Young adulthood and middle age are crucial periods for determining cognitive health in old age. The importance of cardio metabolic risk factors such as obesity and hypertension, smoking and physical inactivity that develop in middle age suggest that preventive approaches are required for target populations in their 40s and 50s, much before they develop dementia. The commonality of dementia risk with cardiovascular and diabetes risk suggests that dementia could be added to present non-communicable disease management programs in primary healthcare and broader public health programs.

## 1. Introduction

According to Hendrie et al. (1) “cognitive health is the development and preservation of the multidimensional cognitive structure that allows older people to maintain social connectedness, an ongoing sense of purpose and the abilities to function independently, to permit functional recovery from illness or injury, and to cope with residual functional deficits.” The main features include mental abilities, acquired skills, and the ability to apply these to complete a purposeful task/activity (2).

Cognitive health involves thinking, learning, and remembering, besides other aspects such as the motor function of making and controlling movements, including balance; emotional function of interpreting and responding to emotions and tactile function like feeling and responding to sensations of touch. Good brain health enables individuals to comprehend their abilities and adjust their cognitive, psychological, emotional, and behavioral functioning according to various life events to cope optimally.

Multiple factors affect the brain’s health, such as age-related changes in the brain, injuries, mood disorders, substance abuse and diseases. While some cannot be changed, evidence exists of many modifiable lifestyle factors: diet and physical activity, social engagement, and cognitive activity, smoking and alcohol consumption which may stabilize or improve declining cognitive function (3). These factors work *via* multiple mechanisms and are thought to either increase or reduce the risk of dementia. Another tool that scientists have proposed is that of cognitive reserve, that is the brain’s capacity to provide a buffer against any brain pathology. Cognitive decline or dementia symptoms may manifest only when there is a more significant threshold/burden of pathology (4).

With fast-evolving evidence on the various dimensions of nutrition and cognitive health covering the human lifespan, there is a need to document the scientific evidence-based data available. So far, the focus has been mainly on the role of nutrition in brain development in the early years. There is a strong emerging need to identify the role of various lifestyle factors, including diet, nutrition, and physical activity, on age-related cognitive decline, which will open the use of new approaches for prevention, treatment or management of age-related disorders and maintaining a good quality of life among older adults.

## 2. Methodology

This review encompasses the role of dietary and nutritional factors on cognitive health through the life course. The scope of this study is to review developments published at the national and international levels, particularly over the last 10 years.

### 2.1. Search strategy

Studies were searched in PubMed over the last 10 years, i.e., 2011–2021. Over 50 keywords, finalized after brainstorming among a few experts, were included in the search, and the investigators repeated the search using all suitable search term combinations. We used MeSH terms for the keywords for searching.

**Figure 1:**
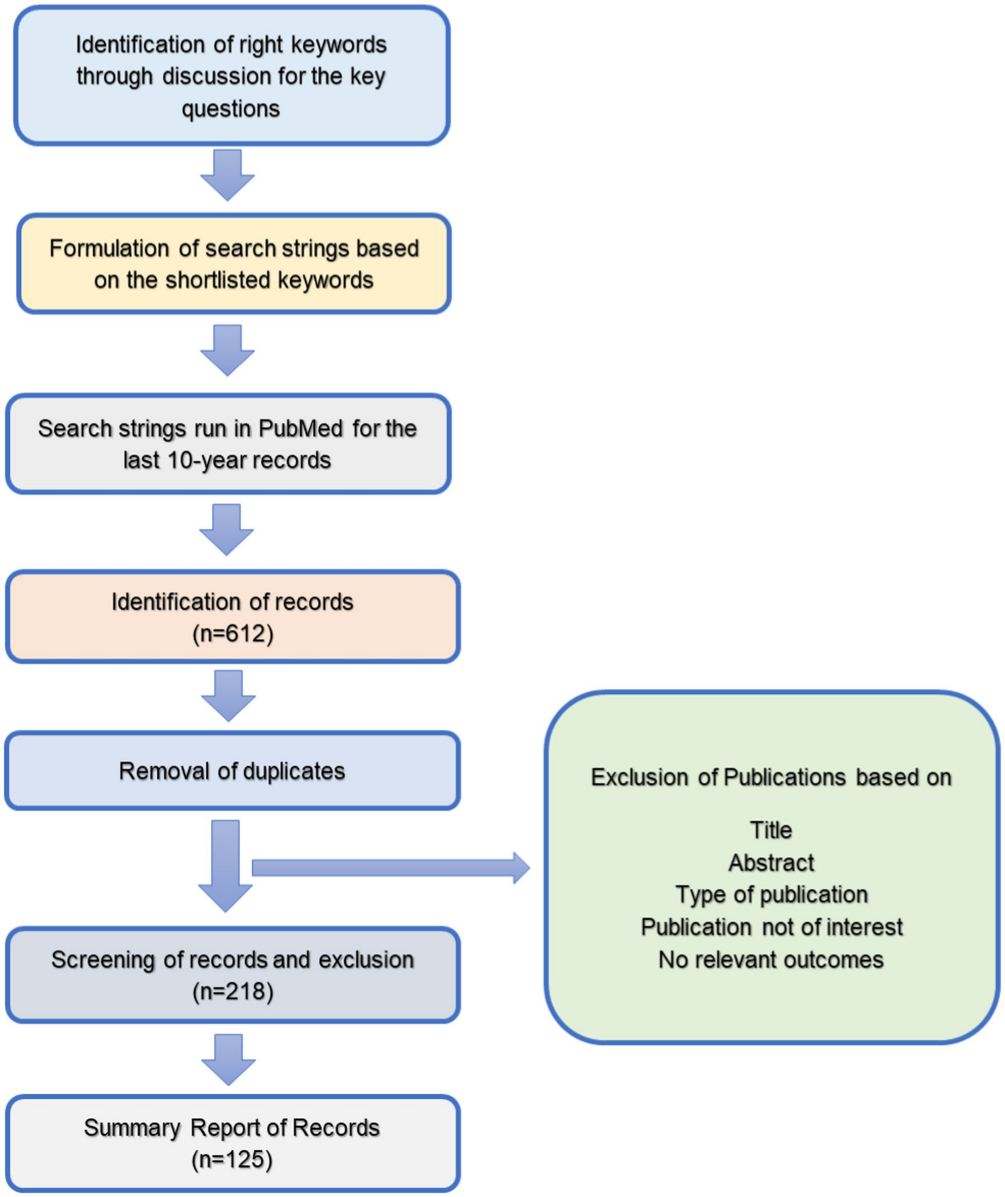
Flow diagram of the search strategy and study selection process.

Studies selected for the present review were observational and intervention studies, review articles, systematic reviews and meta-analysis studies conducted on humans published in MEDLINE. These articles could be accessed with free full text in the English language. Articles that mentioned brain health, cognitive health or cognition and corresponding variables in either the title or abstract were initially included for full-text review. Based on this search, 612 articles were identified. In the second stage, the exclusion criteria, as given below, were applied, and 218 studies were selected. It was decided to examine a maximum of 75–100 full-text articles for in-depth analysis. Hence, finally, 125 full-text articles, after further filtering, were included for review (Figure 1).

The exclusion criteria included data older than 2010, records with words like case reports, rats, sheep, lamb or animal studies etc., in publication title, documents with only basic research or conference proceedings, and records of studies on psychological health like depression, anxiety, emotions, moods etc.

## 3. Nutrition and brain development

Based on brain imaging, it is known that the brain develops throughout childhood and young adulthood. Moreover, different brain structures follow different development and maturation trajectories (5). Genetic predisposition is crucial for brain development. Early childhood experiences have powerful effects on brain function, leading to individual differences that could contribute to behavioral dysfunction and an increased lifetime risk for chronic diseases (6). Nutrition is an essential modulator for early brain development, often over and above the environment to which the child is exposed.

Besides generalized macronutrient under-nutrition, deficiencies of specific nutrients may significantly affect neurodevelopment, with lifelong implications. Several nutrients play an essential role in brain development during pregnancy, including protein, iron, copper, zinc, iodine, folate and certain fats (7). Requirement for these nutrients continues till later life as brain development is an ongoing process (Table 1).

**Table 1:** Critical processes during neurodevelopment affected by specific nutrients.

Cell type	Function	Nutrient example
**Anatomy**		
Neuron	Division (Neurogenesis) Migration Differentiation (Neurite outgrowth; synaptogenesis)	Protein, Carbohydrates, Iron, Copper, Zinc, LC-PUFA, Iodine, Vitamin A, Vitamin B_6_, Vitamin D, Vitamin C
Oligodendrocyte	Myelination	Protein, Carbohydrates, Iron, Iodine, Selenium, Zinc, Vitamin B_6_, Vitamin B_12_
**Chemistry**		
Neuron Astrocyte	Neurotransmitter concentration, receptor, reuptake	Protein, Iron, Iodine, Copper, Zinc, Selenium, Choline, Vitamin B6, Vitamin D
**Physiology and metabolism**		
Neuron Oligodendrocyte	Electrical efficiency	Glucose, protein, Iron, Iodine, Zinc, Choline, Copper

Infancy is also a time of rapid brain growth and development, mainly supported by the baby’s nourishment. Breastfeeding may influence cognitive development through several mechanisms associated with breast milk composition and breastfeeding experience (8). Across all income levels among children and adolescents, breastfeeding is associated with higher performance on intelligence tests (9). The cognitive benefits of breastfeeding reportedly continue into adulthood.

General malnutrition during fetal development and the first few months after birth show life-long deleterious effects on brain development that manifest in learning difficulties (e.g., self-regulation difficulties and lower academic achievement) (10). During early and middle childhood, synapses are rapidly created and later during adolescence are removed selectively; hence, needing a constant supply of nutrients (8). Brain development particularly related to higher cognitive functioning continues through adolescence (11). During adulthood, evidence points to the need for several nutrients to support neuroplasticity and brain performance and to lessen the adverse effects of aging on the brain (12).

### 3.1. Role of nutrition and lifestyle in maintaining cognitive health

In healthy individuals, the decline of cognitive abilities occurs with age and is spread throughout life. The mechanisms which contribute to normal ageing, such as oxidative stress and free radical damage, neuro-inflammation and vascular dysfunction are similar to those contributing to the development of neurological diseases. Due to various genetic or environmental factors, these mechanisms get aggravated in pathological conditions.

Age-related cognitive degeneration is among the leading causes of lost Disability-Adjusted Life Years in people over 65. Addressing the age-related degeneration in neural function is a key to preserving the autonomy and well-being of older people. Besides non-modifiable risk factors, like age and genetic profile which play a significant role in the development of dementia, there is increasing evidence towards the role of modifiable risk factors that enhance or diminish the risk of developing dementia later in life. Factors identified as aggravating risk include depression; type 2 diabetes; midlife hypertension; mid-life obesity; smoking; physical inactivity and low educational attainment (13). Evidence on the role of nutrition in preventing cognitive decline in older adults is now emerging. Although the evidence is not substantial, factors that may reduce dementia risk include vegetable intake, a Mediterranean diet, and increased cognitive activity. There is also some evidence to suggest that events such as the death of a parent early in life and chronic sleep disturbances in middle age may also contribute to an enhanced risk of developing dementia (14).

#### 3.1.1. Macronutrients that affect cognitive health

In the meta-analysis by Coelho-Junior et al. (15) no significant associations were observed between protein intake and global cognition in old age. However, as evident in three studies, there was a positive correlation between memory and protein intake. In only one study, protein intake was positively associated with visuospatial function, verbal fluency, processing speed, and sustained attention (16). A cross-sectional study by Li et al. (13) reported a positive association between protein intake from animal foods, meat, eggs and legumes and cognition.

Various studies on adults and older adults have suggested that a high-fat diet has adverse effects on cognition. A longitudinal study on 6,183 older females in the United States, reported that high amounts of saturated fatty acids were associated with worse cognitive and verbal memory trajectories. In contrast, higher MUFA intake was related to better trajectories (16). A review by Francis and Stevenson (17) reported an association between a high saturated fat, high refined carbohydrate diet and impaired cognitive function. Consumption of a high-fat diet stimulates the hippocampus to produce a neuro-inflammatory response to even a mild immune challenge, resulting in memory deficits (18). A high-fat diet increases the risk of obesity, increased chances to develop diabetes and the development of cognitive deficits and perhaps Alzheimer’s disease (AD). Insulin resistance, impaired glucose metabolism, and type 2 diabetes mellitus are well-known risk factors for AD (19). While data on effect of high protein diets is not consistent, low-fat diets are protective against cognitive decline.

Polyunsaturated fatty acids (PUFAs) regulate the function and structure of neurons, endothelial cells, and glial cells in the brain. Eicosapentaenoic acid (EPA) and docosahexaenoic acid (DHA), the omega-3 fatty acids, also modify neurotransmission, reduce neuro-inflammation and promote neuronal survival and neurogenesis (20). DHA is crucial for neurogenesis and neuronal migration, synaptogenesis, fatty acid composition of membranes, and fluidity, which affect the neurotransmitter systems, particularly the visual system. These areas of the brain regulate attention, inhibition and impulsivity (21). PUFAs have a role in maintaining cognitive function and preventing dementia due to their anti-thrombotic and anti-inflammatory properties and also affect neural processes (22). Cognitive decline in later years is associated with a high energy intake from protein and fat and a low energy intake from carbohydrates, as reported in a retrospective study in China (23). Low dietary consumption of omega-3 PUFAs can also contribute to memory loss (18).

#### 3.1.2. Micronutrients that affect cognitive development, function and decline

Micronutrients help the body to produce enzymes, hormones, and other compounds needed for proper growth and development (24). Vitamin A, iodine and iron deficiencies are the most crucial public health concerns as they significantly impact the health of populations globally particularly young children and pregnant women in low-income countries (24). While the role of B group vitamins in brain development has been researched in recent years, not much evidence has emerged in the last decade on the association of fat-soluble vitamins and cognitive development in early life. Most studies have focused on their role in cognitive decline in later years.

*B-vitamins* are essential for brain development and function through many mechanisms. Venkatramanan et al. (25) emphasized the importance of adequate vitamin B-12 status, particularly during pregnancy and early childhood, as vitamin B-12 has a role in neural myelination, brain development, fetal and child growth.

Studies assessing the association of B vitamins and cognition in older adults were inconclusive (26–28). Only one study on United States adults showed an association of B-vitamins (niacin, folate, B6, and B12) with better cognitive function in midlife. In postmenopausal women free of Mild Cognitive Impairment (MCI), low folate intake may increase the risk of MCI/dementia in later life.

The role of vitamin D in brain health and cognition is emerging with reports of lower serum 25-hydroxyvitamin D (25(OH) D) levels in those with impaired cognitive function and AD than in healthy controls (29, 30). Moreover, low vitamin D levels increased AD risk 7 years later (31). In an 18-week study which compared high-dose vitamin D3 (4,000 IU/day) to a low dose supplement (400 IU/day) in healthy adults, the high-dose supplementation improved only visuospatial memory and no other cognitive domain (32). In a review by Annweiler et al. (33) executive dysfunction was predicted by lower serum 25(OH) D concentrations, while its association with episodic memory was inconclusive (33). A meta-analysis observed cognitive impairment in patients deficient in vitamin D (34). A systematic review by van der Schaft et al. (35) reported that poor outcome in various cognitive tests was linked to a higher dementia incidence risk in patients with low 25 (OH) D levels. Hypovitaminosis D was also associated with a subjective cognitive complaint that predicts cognitive decline and dementia (36).

While many studies show that the intake of antioxidant vitamins (E and C) results in reduced risk of cognitive decline, mixed results are reported. The use of vitamin E and C supplements resulted in a reduced risk of cognitive decline in a prospective cohort in Canada (37). Studies also reported no association (38) or inverse association (39) between antioxidants and cognition. A cross-sectional United States-based study associated a high vitamin E intake with a higher score on verbal memory, immediate recall, and better language/verbal fluency performance (40). A cross-sectional study by Chouet et al. (41) on 192 French older adults reported that higher dietary phylloquinone (vitamin K) was associated with better cognition related behavior among older adults.

Iron is essential for transport of oxygen to all the body’s organs including the brain, as it is a constituent of hemoglobin. Iron deficiency anemia (IDA) is a risk factor for short and long-term cognitive impairment. IDA is associated with poor mental and motor development in infancy and with poor cognition and school achievement during later childhood (8). There is a consensus that prevention is preferable to treatment of iron deficiency and that it is better to protect the brain from suboptimal iron status at an early stage, e.g., in the prenatal period and early infancy (21). Iron deficiency in the brain is associated with disordered neurophysiological mechanisms and hence, compromise motor and cognitive development (e.g., impaired coordination, executive function, attention, and memory) (42).

On the other hand, iron overload in the brain also impairs neurophysiological mechanisms (e.g., enhanced oxidative stress, neuronal cell death) and is associated with decline in motor and cognitive functions (e.g., slow motor function, altered feedback processing and sensitivity, memory loss, and impaired decision-making) (43).

#### 3.1.3. Dietary patterns and food groups

Nutritional components, as well as diet as a composite, affects brain maintenance and function. Healthy diets may protect against dementia and mild cognitive impairment. Smyth et al. (42) have reiterated that a higher intake of healthy foods is a powerful potential method for reducing the global burden of cognitive decline. Wright et al. (44) demonstrated that better cognitive performance, particularly in verbal retention and memory is associated with a higher diet quality irrespective of race and poverty status. There is evidence to support the ‘whole diet approach’ theory, i.e., a balanced diet, as a whole, rather than single nutrients is beneficial for brain health. Specific dietary patterns that may prove more valuable than consuming individual food/food groups include the Mediterranean diet, the Nordic Diet, and the DASH Diet.

The Mediterranean diet refers to the diet of people in Greece, Spain, France, Italy, Egypt, Algeria, and Libya. The key features of this diet are that unrefined carbohydrates and starches are consumed in large amounts along with cheese, yoghurt, fruits, and vegetables. Chicken, fish, and eggs are consumed a few times in a week, while red meat intake is not more than a few times in a month. Fat content varies from 28 to 40 percent, mainly from an unsaturated source, olive oil (45). Studies have shown that this diet is linked to a low risk of cognitive decline (46–48), lower prevalence of dementia, depression, (49, 50) and reduced risk of Alzheimer’s disease (48). A PREDIMED sub-study assessed cognitive performance at baseline and after 4 years. There was improvement in cognitive function in subjects on the Mediterranean diet and a decline in cognitive function among those on the control diet (51). There is evidence of a moderate protective effect of the Mediterranean diet against cognitive decline and Alzheimer’s disease based on large longitudinal observational studies (48, 52, 53). Another systematic review by van de Rest et al. (54) reported that greater adherence to a Mediterranean diet is associated with lesser cognitive decline, dementia, or Alzheimer’s disease, as evident in several cross-sectional, longitudinal studies, trials, and meta-analyses.

The Nordic Diet is based on the types of food consumed in Scandinavian countries (55). The emphasis is on foods and nutrients such as fruits and vegetables, fish, canola oil, and several types of meat. A 4-year study to examine the associations of Nordic Diet with cognitive function was conducted on 1,140 men and women with normal cognition. It revealed that subjects who followed the guidelines of the Nordic Diet had enhanced levels of cognitive functioning compared to baseline (56).

The Dietary Approaches to Stop Hypertension (DASH) Diet is characterized by low sodium content and small portion sizes, which have significant health benefits. The DASH diet improved cardiovascular risk factors and had greater beneficial effects in subjects with an increased cardio metabolic risk (57). MIND or Mediterranean-Dietary Approaches to Stop Hypertension (DASH) Intervention for Neurodegenerative Delay includes specific guidelines beneficial for brain health. The foods included in the MIND diet are antioxidant-rich to enhance cognition; green leafy vegetables to prevent cognitive decline (55), and blueberries to improve memory (58–60) fish to help maintain cognitive function due to high amounts of EPA and DHA present (61). More research is warranted to establish the role of this diet in maintaining brain health.

Asian plant-based dietary patterns are based on foods like whole grains, soy, green leafy and other vegetables, green tea, mushrooms, and seaweed. Evidence strongly suggests that these dietary patterns are associated with a lower risk of cognitive impairment, a slower rate of cognitive decline and better logical memory or higher global cognitive assessment scores (62).

According to van de Rest et al. (57) other healthy dietary patterns derived both *a priori* (e.g., Healthy Diet Indicator and Healthy Eating Index) and *a posteriori* (e.g., factor analysis, cluster analysis, and reduced rank regression), were also associated with reduced cognitive decline and/or a reduced risk of dementia (54).

#### 3.1.4. Importance of breakfast

Literature has addressed the effects of consuming breakfast on cognition. Breakfast composition has a profound impact on multiple cognitive domains: attention capacity (63); processing speed (64); working memory (65); immediate recall, delayed recall, recognition (66). Adolphus et al. (67) reported that breakfast consumption in children and adolescents (4–18 years), compared to fasting, had a short-term (same morning) positive effect on cognition tasks requiring attention, executive function, and memory. In adults over 18 years, breakfast consumption showed a small but robust advantage for memory (particularly delayed recall), but the effect on attention and motor and executive function was not well established. No effects of breakfast on language were evident (68).

#### 3.1.5. Food group intake

The role of different food groups on brain function and cognitive decline is well documented. Consumption of refined cereals and grains was associated with worse cognitive function and decline (69), while unrefined cereals and whole-grain consumption was associated with better cognitive function (47, 70, 71). An inverse relationship was observed between refined carbohydrate intake and non-verbal intelligence (72). As seen in both cross-sectional and longitudinal studies, a positive association was noted between fish intake and cognition (51, 69, 73, 74). Fish consumption lowered the risk of cognitive decline, MCI, and dementia (69, 70, 73, 74). One study reported better attention, visual memory, episodic verbal memory, working memory and executive function in both adults and older adults who consumed fish, but worse cognitive and executive function was reported in subjects on the consumption of red meat (74). Dairy consumption (high-fat milk) was associated with worse cognitive function (74) and cognitive decline (69), while no association was seen with cheese (75) and ice-cream intake (69). In addition, foods like avocados (76), berries (60) and extra-virgin olive oil (77) were associated with the delay of cognitive decline (78).

In cross-sectional as well as longitudinal studies, better cognitive function and lesser cognitive decline were seen in subjects who consumed plant-based foods (47, 73–75) olive oil (51, 74, 76) legumes (71) and walnuts (72, 74,78) Green leafy vegetable consumption did not lower the risk of cognitive impairment (75). Fruits, berries, potatoes and vegetable consumption were not associated with better cognitive function (51, 70, 74, 77, 78). The effects of a plant-based diet reviewed by Medawar et al. (79) reported the impact on cognition/cognitive processes, brain activity for language and empathy-related tasks, emotional health, and personality traits. Rajaram et al. (62) also reported that consumption of citrus fruits, grapes, berries, nuts, green tea, cocoa and coffee improved specific cognitive domains, especially the executive functions. However, they established no causal relationship between the use of a plant-based diet and its putative effects on cognitive, mental, and neurological functions. Participants in the Mediterranean diet plus nuts group improved in the memory composite compared to the control diet group (80). Two observational studies, the Doetinchem Cohort (81) and the Nurses’ Health Study (82), reported that long-term nut consumption was related to better overall cognition at an older age but not to cognitive decline during follow-up for 5 to 6 years.

Alcohol intake was not associated with cognitive function (70, 73, 74, 80, 81) except better global cognition was associated with wine intake in community-dwelling residents of Spain (51) No association with cognitive decline was seen with the intake of spirits/beers in the longitudinal study by Shakersain et al. (69). No association with cognitive decline or impairment was observed in longitudinal studies conducted on the intake of sugar/fruit juices (69), sweetened beverages, sodium intake (71), processed and fast/fried food, and sweets and pastries (75), animal-based cooking fat (83).

A systematic review to evaluate the impact of healthy diet consumption among children and adolescents on executive functioning reported that among the ten studies examined, there was a positive association between healthier foods (e.g., whole grains, fish, fruits, and vegetables) and executive function (84). In contrast, intake of less-healthy snack foods, sugar-sweetened beverages and red/processed meats were associated with poor executive functioning.

Certain herbs that may prove beneficial in enhancing cognition or delaying cognitive decline include *Ashwagandha*, turmeric, *Brahmi* etc. In a systematic review, the extract of *Ashwagandha (Indian Ginseng/Winter Cherry)* corrected mild cognitive impairment and enhanced executive functions in adults with MCI (85). Curcumin in *turmeric* reduces oxidative damage and improves cognitive functions related to senescence. It also binds with β-amyloid plaques, inhibits its aggregation, and is beneficial in Alzheimer’s disease. An RCT demonstrated that 400 mg/day of curcumin improved sustained attention and working memory functions in adults over 60 (86). *Brahmi (waterhyssop/thyme-leafed gratiola/Indian pennywort)* is commonly used as a memory enhancer.

#### 3.1.6. Other dietary components

Polyphenols are secondary metabolites of plants and comprise flavonoids, lignans, stilbenes, coumarins and tannins. They are present abundantly in colorful fruits (berries, grapes, and tomatoes), vegetables, tea, spices, herbs, and olive oil. They contribute to brain health similar to antioxidants by regulating oxidative stress and mediating anti-inflammatory mechanisms (87). The extensively studied group in relation to brain health, under polyphenols is flavonoids. There is evidence for established associations between flavonoids and delayed cognitive decline (88), and enhanced language and verbal memory tasks (46). Cocoa flavonoids (in dark chocolate) enhance cognition (87, 88) however, a study on participants with cognitive impairment involving cocoa flavonoids was inconclusive (89). An RCT conducted on healthy adults aged 50–69, reported that a high cocoa flavanol-containing diet enhanced gyrus function after 3 months (90) and was associated with a 41% lower risk of cognitive decline (91).

In the review by Jirout et al., (10) high levels of carotenoids present in leafy vegetables were associated with higher scores on tests in the visual–spatial domain. Lutein, one of the three major dietary carotenoids present in the brain (92), is of functional importance to cognition and infant brain development. It is also associated with density of the macular pigment which interacts with cognitive functioning (93). Interestingly, lutein concentration was higher in the brains of children than in adults and was clearly related to cognition, i.e., executive function, language, learning, and memory (93) and improved the speed of temporal processing in young adults (94).

There are limited studies to elucidate the role of caffeine in memory and cognition enhancement. High serum levels of caffeine delayed dementia progression in a case–control study on 124 older persons with MCI (95). However, another study did not find any association with risk of cognitive impairment, overall dementia, or AD (96). Lesser cognitive decline among coffee consumers was seen in a longitudinal study but this decline was not dose related (97).

Soy isoflavones include genistein and diadzein, and their effects on cognition are variable and inconclusive (98), with an overall absence of adverse events (99). An initial positive effect in adults appears to reverse in older women; in men, the data are even more inconclusive (98).

In the Doetinchem Cohort Study, which included 2,613 participants 43–70 years old, higher consumption of allium (onion, garlic, and leek) was associated with poorer scores on cognitive flexibility and speed of cognitive processes in the cross-sectional analyses. However, in the longitudinal data, allium consumption was not seen to be associated with cognitive decline (81).

### 3.2. Microbiome-gut-brain Axis

According to Korecka and Arulampalam (100) the term “gut microbiome” refers to “the complex ecosystem of bacteria that colonize the gut, including their genes, proteins, and metabolites” (101). Although evidence on how the gut microbiome may modulate brain development is less, immune signaling is likely to play a critical role (102). The benefits of human-microbe symbiosis are now known to extend to human mental health, with growing evidence that the gut–brain axis plays a crucial role in maintaining brain health *via* bidirectional communication between the microbes and the brain (101, 102). It also affects human behavior and the pathophysiology of mental illnesses (101, 103).

The two-way interaction between the gut and the brain, i.e., the microbiome-gut-brain axis is now well recognized. Emerging evidence has revealed the importance of the gut microbiome in this bidirectional communication system, i.e., enabling the gut microbes to communicate with the brain and the brain with the gut (102).

The gut microbiome is highly influenced by negative external lifestyle factors, such as poor diet, sleep deprivation, circadian rhythm disturbances, chronic noise and sedentary behavior, which are also important risk factors for the development of Alzheimer’s and other non-communicable diseases (104). Evidence for the beneficial effects of dietary fibers and probiotics through the management of gut microbes is strongly emerging. Several published research have shown the effect of intestinal dysbiosis caused by dietary changes, the use of antibiotics, non-steroidal anti-inflammatory drugs, and the presence of pathogenic microorganisms on the brain’s cognitive functions (104).

Immune cells, cytokines, and chemokines are the microbiome’s mechanisms for interacting with the brain, regulating brain processes and vice versa (99, 105). The gut microbiota may modulate brain function and development through such immune signaling as well as endocrine and neural pathways. Nutritional components may influence each communication pathway (102). Conversely, the brain may impact the gut through neurotransmitters that affect immune function and alter cortisol levels, intestinal motility, and permeability. The gut microbiota changes dynamically across the lifespan, establishing their relationship with the host at critical periods during infancy, adolescence, and ageing. There is an increased vulnerability to external insults at these time windows, resulting in greater susceptibility to brain disorders. Disturbances of the developing gut microbiota early in life can impact neurodevelopment significantly and lead to adverse mental health outcomes later in life.

Similarly, the microbiota may contribute to ageing and the developmental course of neurodegenerative disorders The gut microbiota also regulates key central neurotransmitters by altering levels of precursors—the inhibitory neurotransmitter γ-aminobutyric acid is produced by *Lactobacillus* and *Bifidobacterium* species; noradrenaline (norepinephrine) is produced by *Escherichia*, *Bacillus* and *Saccharomyces* spp.; *Candida*, *Streptococcus*, *Escherichia*, and *Enterococcus* spp. produce serotonin; *Bacillus* produce dopamine, and certain *Lactobacillus* spp. can produce acetylcholine (106, 107). These microbially synthesized neurotransmitters can cross the intestines mucosal layer and possibly mediate brain physiological events (106). Short-chain fatty acids, including propionate, butyrate and acetate, are metabolic products of gut microbial activity and may exert central effects directly or indirectly (108–110).

Majority of the evidence for the involvement of the gut microbiota in cognition is provided by animal experiments on induced infections (111) antibiotic and dietary manipulations (112, 113). and probiotic interventions (114).

Dietary Recommendations for Brain Health*

- A Mediterranean-like healthy diet which contains fruits, vegetables, legumes (e.g., lentils, beans), nuts and whole grains (e.g., unrefined maize, millet, oats, wheat, brown rice).
- At least 400 g (five portions) of fruits and vegetables daily. Potatoes, sweet potatoes, and other starchy root vegetables are not included in this category of fruits or vegetables.
- Less than 10% of total energy intake from free sugars. This is equivalent to 50 g (or 10 teaspoons) for a person of healthy body weight consuming approximately 2000 calories per day. Ideally consume less than 5% of total energy intake (i.e., 25 g) for additional health benefits. Most free sugars are added to foods or drinks by the manufacturer, cook or consumer, and can also be found naturally present in honey, syrups, fruit juices and fruit juice concentrates.
- Less than 30% of total energy intake from fats. Unsaturated fats (in fish, avocado, nuts, sunflower, canola and olive oils) are preferable to saturated fats (fatty meat, butter, palm and coconut oil, cream, cheese, ghee) and trans-fats of all kinds, including industrially produced trans-fats (in processed food, fried foods, cookies, biscuits, wafers, margarines and spreads) and ruminant trans-fats (in meat and dairy foods from ruminant animals, such as cows, sheep, goats, and others). Reduce the intake of saturated fats to less than 10% of total energy intake and trans-fats to less than 1% of total energy intake.
- Less than 5 g of iodized salt (approximately 1 teaspoon) per day.

[Source: (115).]

*For Adults with normal cognition or MCI. Several studies have reported improvements in cognitive function on the administration of probiotics, particularly in those with MCI (116). However, Louzada et al. (117) reported no effect on cognition on the administration of a symbiotic.

### 3.3. Nutritional interventions to reduce risk of cognitive decline and dementia

World Health Organization (115) has given detailed guidelines for reducing cognitive decline and dementia risk. The dietary approaches associated with better cognitive function include the Mediterranean diet (52, 118). Dietary Approaches to Stop Hypertension (DASH) (55) and the brain health-specific Mediterranean-DASH Intervention for Neurodegenerative Delay (MIND) diet. WHO’s recommendations for the diet are based on the Mediterranean-like diet.

There is a significant association between excess fat mass and cognitive impairment. Therefore, WHO (115) has outlined recommendations for weight management, including dietary advice and physical activity. Stress is also laid on the appropriate management of hypertension, dyslipidemia and diabetes.

Multiple factors affect brain health from conception till old age: modifiable and non-modifiable. The modifiable factors include diet, physical activity, social engagement, cognitive activity, smoking and alcohol consumption. Strategies to promote brain health throughout life should target individuals at each phase of life to adopt a healthy lifestyle (diet and physical activity), be engaged in cognitively stimulating activities and be socially active.

## 4. Conclusion

Good brain health enables an individual to comprehend their abilities and adjust their cognitive, psychological, emotional, and behavioral functioning according to various life events to cope optimally.

In addition to generalized macronutrient under-nutrition, individual nutrient deficiencies may substantially affect brain development and subsequent cognitive health. Several micronutrients like B group vitamins and iron play a crucial role in cognitive health. High protein and low-fat diets are protective against cognitive decline.

A salient potential approach for lowering the global burden of cognitive decline is to ensure a higher diet quality by ensuring a higher intake of healthy foods. Evidence supports the ‘whole diet approach’ theory that a balanced diet, rather than single nutrients, benefits cognitive health. Mediterranean, Nordic, DASH, and MIND diets are linked with a low risk of cognitive decline and dementia. A balanced diet should be encouraged *via* nutrition counseling in early adult life and regular physical activity to promote a healthy lifestyle.

In recent years, considerable information is available on the relationship between the gut microbiome and brain function through the gut-brain axis. The gut microbiome is highly influenced by negative external lifestyle factors, such as poor diet, sleep deprivation, circadian rhythm disturbances, chronic noise, and sedentary behavior, which are also important risk factors for the development of NCDs and Alzheimer’s. Data on the beneficial effects of dietary fibers and probiotics through the management of gut microbes is strongly emerging.

The role of nutrition and lifestyle factors in cognitive development, function and decline is an area garnering increasing attention in recent years. Several decades ago, some pioneering research was carried out to establish brain growth patterns and the influence of genetic and environmental factors on these. However, there is a dire need to examine the effect of the epidemiological and societal transition on cognitive health.

Moreover, the factors that have been derived are based on observational studies, and unidentified confounders may bias the results. These studies often lack consistency and detail in the description or categorization of lifestyle factors and sometimes in the measured cognitive outcomes. Longer duration longitudinal studies or cohorts are needed to get better insights into the lifestyle factors that affect cognition.

While a significant number of research are now focusing on old age, the period of adulthood is overlooked. Young adulthood and middle age are crucial periods for determining cognitive health in old age. Moreover, many existing studies are cross-sectional, so getting a life course perspective is difficult. It is therefore vital to conduct large longitudinal studies and studies established cohorts to examine the influence of environmental exposures on early life brain development and cognitive health through the life cycle, including adulthood.

Unlike non-communicable diseases like cardiovascular disorders, diabetes and cancer, there is a dearth of epidemiological data on cognitive impairment and dementias. Large national surveys should include these disorders to estimate their prevalence and related epidemiological data. In India, the Longitudinal Ageing Study in India (119), conducted by the International Institute of Population Studies and facilitated by the Ministry of Health and Family Welfare, did capture the reported prevalence of Alzheimer’s disease among 44 to 99-year-olds as only 0.4%. However, these were previously diagnosed cases, so there is a need to include screening and assessment for cognitive dysfunction to detect undiagnosed cases in such surveys.

Several countries have now acknowledged the importance of focusing on declining cognitive health among older adults and the need for preventive strategies targeting dementia. The importance of cardio-metabolic risk factors that develop in middle age, such as obesity and hypertension as well as smoking and physical inactivity, suggest that preventive approaches are required for target populations in their 40s and 50s, much before they develop dementia. The clear overlap with cardiovascular and diabetes risk suggests that dementia should be included in current non-communicable disease (NCD) management programs at primary health care level as well as broader public health programs.

The challenge for nutritionists is integrating existing scientific knowledge, and further advancing applied research on effective ways to attain and maintain optimal cognitive function throughout life. Of potentially far-reaching consequences is the concept that early life nutrition may program metabolic functions, leading over time to an increasing imbalance and promoting the emergence of disease states. A dietary approach as well as macronutrient and micronutrient intake that has protective effects against CVD is most likely to be effective against neurodegenerative disorders too.

## Author contributions

SP and MS were responsible for the conceptualization of the manuscript, compiling the data, and writing the manuscript. SP and BG were responsible for reviewing and editing the manuscript. All authors contributed to the article and approved the submitted version.

## Conflict of interest

The authors declare that the research was conducted in the absence of any commercial or financial relationships that could be construed as a potential conflict of interest.

## Publisher’s note

All claims expressed in this article are solely those of the authors and do not necessarily represent those of their affiliated organizations, or those of the publisher, the editors and the reviewers. Any product that may be evaluated in this article, or claim that may be made by its manufacturer, is not guaranteed or endorsed by the publisher.
